# Epigenetic regulation of caloric restriction in aging

**DOI:** 10.1186/1741-7015-9-98

**Published:** 2011-08-25

**Authors:** Yuanyuan Li, Michael Daniel, Trygve O Tollefsbol

**Affiliations:** 1 Department of Biology, University of Alabama at Birmingham, 1300 University Boulevard, Birmingham, AL 35294, USA; 2 Center for Aging, University of Alabama at Birmingham, 1530 3rdAvenue South Birmingham, AL 35294, USA; 3 Comprehensive Cancer Center, University of Alabama at Birmingham, Birmingham, 1802 6th Avenue South, AL 35294, USA; 4 Nutrition & Obesity Research Center, University of Alabama at Birmingham, 1675 University Boulevard, Birmingham, AL 35294, USA; 5 Diabetes Comprehensive Center, University of Alabama at Birmingham, 1825 University Boulevard, Birmingham, AL 35294, USA

**Keywords:** caloric restriction, epigenetic, aging

## Abstract

The molecular mechanisms of aging are the subject of much research and have facilitated potential interventions to delay aging and aging-related degenerative diseases in humans. The aging process is frequently affected by environmental factors, and caloric restriction is by far the most effective and established environmental manipulation for extending lifespan in various animal models. However, the precise mechanisms by which caloric restriction affects lifespan are still not clear. Epigenetic mechanisms have recently been recognized as major contributors to nutrition-related longevity and aging control. Two primary epigenetic codes, DNA methylation and histone modification, are believed to dynamically influence chromatin structure, resulting in expression changes of relevant genes. In this review, we assess the current advances in epigenetic regulation in response to caloric restriction and how this affects cellular senescence, aging and potential extension of a healthy lifespan in humans. Enhanced understanding of the important role of epigenetics in the control of the aging process through caloric restriction may lead to clinical advances in the prevention and therapy of human aging-associated diseases.

## Introduction

Aging and its direct consequences, such as degenerative diseases and even death, are inevitable; however, scientific advances in understanding basic aging mechanisms have made it much more feasible to postpone aging processes and to increase the human lifespan using clinical approaches. Current studies using model organisms indicate that aging processes can be manipulated by many interacting factors which include, but are not limited to, geneticnutritional and pharmacological interventions [1-3]. Studies of monozygotic twins, who share the same genotype and often present many phenotypic differences [4-7], indicate that external environmental factors contribute to interindividual differences such as susceptibility to disease and the potential to live longer.

Dietary control, as a major environmental factor, has a profound effect on many aspects of health, including aging, and caloric restriction (CR) is by far the most effective environmental manipulation that can extend maximum lifespan in many different species [8,9]. In fact, the remarkable effect of CR on aging was first defined in experimental animal models in which McCay *et al. *[10] discovered that rats fed a calorie-restricted diet lived longer than control rats fed a regular diet. Since then, numerous research findings have revealed effects of CR on lifespan interference among diverse, but not all eukaryotes, including yeast, worms, flies, fish and even mammals [11-13]. In most rodent CR studies, the limitation of total calories derived from carbohydrates, fats or proteins to a level 25% to 60% below that of control animals fed *ad libitum*, while containing all essential nutrients [14-16], can result in a significant lifespan extension in 50% of rodents [17-21]. In addition to increasing lifespan in rodents, CR has also been shown to delay a wide range of aging-associated diseases, such as cancer, diabetes, atherosclerosis, cardiovascular diseases and neurodegenerative diseases in higher mammals, such as nonhuman primates and humans [13,22-24] (Table 1). The incidence of disease increases with age and is a fundamental contributor to mortality. Thus, CR may affect aging processes by favorably influencing broad aspects of human health.

**Table 1:** Summary of aging-related diseases affected by caloric restriction in experimental animal models and clinical trials^a^

Diseases	Findings	Rodents	Nonhuman primates	Humans	References
Cancer	CR prevents a broad range of cancer incidences, including breast and gastrointestinal cancer.	Y	Y	Y/?	[17,13,23]
Diabetes	CR improves glucose homeostasis and prevents diabetes.	Y	Y	Y	[18,13,23,24]
Cardiovascular diseases	CR lowers blood pressure and favorably alters lipid profile, resulting in significantly reducing the risk of cardiovascular disease and related complications.	Y	Y	Y	[19,13,22-24]
Neurodegenerative diseases	CR reduces aging-associated neuronal loss and neurodegenerative disorders such as Parkinson's disease and Alzheimer's disease.	Y	Y	Y/?	[20,13,23]
Immune deficiencies	CR delays the onset of T-lymphocyte-dependent autoimmune diseases.	Y	Y/?	Y/?	[21]

^a^CR, caloric restriction; Y, CR has effects on relevant physiological changes; Y/?, not resolved or not reported.

Numerous studies suggest that the effects of CR in the prevention of the onset of many aging-related degenerative diseases occur through various molecular mechanisms, including reduction of oxidative stress or regulation of metabolic pathways during the progression of aging [14,25,26]. However, the precise mechanisms of CR-induced longevity are not very well understood. Recently, epigenetic mechanisms have received considerable attention due to the unique role of interactions with multiple nutritional factors and the aging processes. Epigenetic control is believed to dynamically regulate gene expression by mechanisms other than changes in the DNA sequence. This primarily affects two epigenetic codes: DNA methylation and histone modification [27-29]. Recent evidence suggests that DNA methylation status changes in specific gene loci may play an essential role in CR-dependent aging postponement and longevity [30,31]. More concrete evidence has emerged, most notably the discovery of silent mating type information regulation 2 homolog 1 (Sirtuin 1), a nicotinamide adenine dinucleotide (NAD^+^)-dependent histone deacetylase (HDAC), since Sirtuin 1 activity has been linked to the control of lifespan in response to CR both *in vivo *and *in vitro *[32-36]. Although studies of the characterization and function of epigenetic modifications in CR-associated longevity are just emerging, a better understanding of this complex interaction provides promising clinical opportunities for the prevention of human aging and degenerative diseases that often accompany the aging process.

### DNA methylation affects aging during caloric restriction

DNA methylation is one of the most important epigenetic modifications. It provides a stable and heritable component of epigenetic regulation. DNA methylation primarily occurs on cytosine residues of CpG dinucleotides, which are frequently clustered into CpG islands at the regulatory sites of gene promoter regions. The amount of DNA methylation in a gene control region generally inversely correlates with gene activation [37,38]. The methyl groups on CpG dinucleotides can recruit multiple transcriptional complex proteins, including methylation-sensitive transcription factors and methyl-binding proteins that are often associated with gene silencing [39]. Therefore, DNA methylation plays an important role in the regulation of gene expression, maintenance of DNA integrity and stability in many biological processes, such as genomic imprinting, normal development, cell proliferation and aging [40-42]. The patterns of DNA methylation are dynamically mediated by at least three independent DNA methyltransferases (DNMTs): DNMT1, DNMT3a and DNMT3b. DNMT1 performs a maintenance function during cell division, while DNMT3a and DNMT3b act as *de novo *methyltransferases after DNA replication by adding a methyl moiety to the cytosine of CpG dinucleotides that have not previously been methylated [43-47].

During aging processes, there is a progressively reduced capability for homeostasis and loss of chromatin integrity, predominantly due to aberrant gene expression [48]. DNA methylation regulation plays a crucial role during aging processes. Age causes a dramatic change in the distribution of 5-methylcytosine (the product of DNA methylation) across the genome. This leads to a decrease in global DNA methylation [49-54]. Although genome-wide levels of methylation decrease with aging, the promoter regions of many specific genes tend to switch from unmethylated to methylated status, resulting in gene silencing, which may include promoters of several tumor- and/or aging-related genes, such as *RUNX3 *and *TIG1 *[53,55] (Table 2). These findings suggest an essential role of aging-associated DNA methylation changes in the regulation of aging-related diseases such as cancer.

**Table 2:** Selected genes regulated by epigenetic factors during caloric restriction^a^

Genes	Gene functions in aging	Epigenetic regulation	CR effects	References
*p16^INK4a^*	Tumor suppressor gene that inhibits cell cycle and accumulates during aging	DNA methylation, histone acetylation (mediated by SIRT1 and HDAC1) and histone methylation	Downregulation	[31,84]
*p53*	Tumor suppressor gene that induces cell cycle arrest, apoptosis and senescence; increased *p53 *promotes aging	Histone acetylation (mediated by SIRT1)	Downregulation	[88-90]
*H-ras*	Oncogene that accelerates aging	DNA methylation	Downregulation	[30]
*RUNX3*	Transcription factor that plays important roles in development; increases methylation with aging	DNA methylation	Up regulation	[53,55]
*Foxo*	Forkhead transcription factors that control various biological functions and involve SIRT1-related longevity	Histone acetylation (mediated by SIRT1)	Downregulation	[91,92]
*Ku70*	A component of the NHEJ pathway for DSB repair that regulates apoptosis and DNA repair during aging	Histone acetylation (mediated by SIRT1)	Downregulation	[99,100]
*PGC-1α*	Regulates mitochondrial function and glucose homeostasis and interacts with SIRT1 to regulate glucose metabolism during CR	Histone acetylation (mediated by SIRT1)	Upregulation	[34,83,93,94]
*hTERT*	Tumor promoting gene; increased *hTERT *expression is correlated with telomerase activation and aging delay	Histone acetylation (mediated by HDAC1) and histone methylation	Upregulation	[31]

^a^CR, caloric restriction; *hTERT*, human telomerase reverse transcriptase; HDAC1, histone deacetylase 1; SIRT1, Sirtuin (silent mating type information regulation 2 homolog) 1; NHEJ, non-homologous end joining; DSB, DNA double-strand break.

The evidence suggests that the biological effects of CR are closely related to chromatin function [56]. In fact, acting as an important environmental intervention, CR is speculated to exert its aging-delaying effect through its capacity to increase genomic stability. Reversal of aberrant DNA methylation during aging is believed to be the most effective mechanism for CR to maintain chromatin function and subsequently influence aging processes.

**Figure 1:**
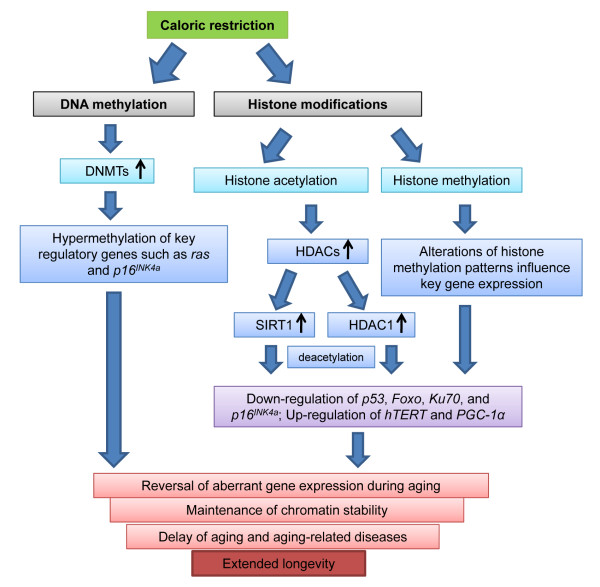
**Caloric restriction regulates epigenetic pathways**. Caloric restriction (CR) influences epigenetic processes via two primary mechanisms: DNA methylation and histone modification. DNA methylation regulation during CR involves DNMT activation, resulting in silencing the expression of target genes such as *p16^INK4a ^*and *Ras *due to hypermethylation of these genes. CR-induced histone remodeling primarily includes histone acetylation and methylation. Deacetylation effects due to activation of SIRT1 and HDAC1 by CR lead to expression changes of key genes such as *p53*, *Foxo*, *Ku70*, *PGC-1α *and *p16^INK4a^*. Histone methylation also plays a role in the regulation of key gene expression, including *hTERT *and *p16^INK4a^*. As a result, epigenetic regulation actively reverses aberrant gene expression during CR, which contributes to CR-associated aging delay and lifespan extension.

As discussed previously, two major changes in DNA methylation occur during aging progression. These changes involve globally decreased but locally increased DNA methylation status. Interestingly, CR is likely to recover these aging-induced aberrant DNA methylation patterns, but by specific loci control rather than globally [57] (Figure 1). Studies of the comparison of DNA methylation levels in pancreatic acinar cells between CR-fed rats and control rats fed *ad libitum *suggest that CR increased the methylation level of proto-oncogenes such as Ras [30] (Table 2). A hypermethylated gene promoter will often be recognized by transcriptional repressor complexes, leading to silencing the expression of these oncogenes, which contributes to the effects of CR on cancer prevention. Although the majority of CR research has been based on experimental animal studies, we have established an *in vitro *mammalian cellular system to mimic CR-controlled longevity by the reduction of glucose, the main caloric resource in cell culture medium [31]. In our current studies of human cells, DNA hypermethylation of an *E2F-1 *binding site was found in the promoter of the *p16^INK4a ^*gene, an important tumor suppressor and aging-associated gene. This DNA hypermethylation of the *E2F-1 *binding site blocks access of *E2F-1 *(an active transcription factor of *p16^INK4a^*) to the *p16^INK4a ^*promoter, resulting in *p16^INK4a ^*downregulation, which contributes to CR-induced lifespan extension (Table 2 and Figure 1). In this regard, there is a strong tendency for the DNA methylation pathway to predominately control key cancer-related genes during CR, suggesting a close connection between aging and cancer.

On the basis of the preceding discussion, we confirm that DNMTs play a crucial role in maintaining or rewriting DNA methylation profiles. Consistently, DNMT1 activity is significantly elevated in response to CR to correct the decreased methylation level during aging [31]. Further studies have also indicated that CR-caused Dnmt3a level changes in the mouse hippocampus may benefit mouse brain function during aging [58]. Both DNMT1 and DNMT3b play a critical role in regulating cellular senescence in human stem cells [59]. Therefore, it is highly possible that CR modulates DNA methylation, depending on expression levels and/or enzymatic activities of individual DNMTs (Figure 1).

Because of the critical roles of DNMTs in the control of aging and aging-associated diseases such as cancer and DNMT inhibitors such as azacitidine (5-azacytidine) and decitabine (5-aza-2'-deoxycytidine) have been widely used for cancer treatment in both experimental studies and clinical trials [60] (Table 3). Moreover, some bioactive food components with DNMT inhibition properties, such as green tea polyphenols and soybean genistein, have shown cancer prevention and inhibition activities by reducing DNA hypermethylation of key cancer-causing genes [61-63] (Table 3). These are important and encouraging findings that imply the potential translation of these bioactive dietary compounds into intervention targets and strategies for the prevention and treatment of human cancer.

**Table 3:** Epigenetic clinical trials for aging-related degenerative diseases^a^

Drugs	Epigenetic effect	Description	Clinical trials	References
Azacitidine	DNMT inhibitors	5-azacytidine; a chemical analogue of cytidine that affects DNA methylation as a false substrate	Phases I, II and III: myelodysplastic syndromes such as leukemia	[60]
Decitabine	DNMT inhibitors	5-aza-2'-deoxycytidine, a chemical analogue of cytidine that affects DNA methylation as a false substrate	Phases I, II and III: myelodysplastic syndromes such as leukemia, cervical, and non-small-cell lung cancer	[60]
Depsipeptide	HDAC inhibitors	Cyclic tetrapeptide	Phases I and II: hematological tumors such as leukemia and lymphoma	[78,79]
Phenylbutyrate	HDAC inhibitors	Aliphatic acid	Phases I and II: hematological tumors such as leukemia and lymphoma and colorectal cancer	[78,79]
Valproic acid	HDAC inhibitors	Aliphatic acid	Phase I: hematological tumors such as leukemia and lymphoma	[78,79]
Suberoylanilide hydroxamic acid	HDAC inhibitors	Hydroxamic acid	Phases I and II: hematological tumors, such as leukemia and lymphoma, solid tumors	[78,79]
Resveratrol	SIRT1 activator	A natural compound enriched in grapes and red wine	Phase I and II: diabetes, obesity, Alzheimer's disease and cancers	[118,119]
Genistein	Inhibitor of both DNMTs and HDACs	Active epigenetic diet found in soybean products	Preclinical: diabetes and cancer	[61,63,122,123]
EGCG	Inhibitor of both DNMTs and HDACs	Active epigenetic dietary compound enriched in green tea	Phase I: diabetes, cardiovascular disease and cancer	[61,62,124,125]
Sulforaphane	HDAC inhibitor	Active epigenetic dietary compound enriched in broccoli sprouts	Preclinical	[80,121]

^a^DNMT, DNA methyltransferase; HDAC, histone deacetylase; SIRT1, Sirtuin (silent mating type information regulation 2 homolog) 1; EGCG, epigallocatechin gallate.

Since restricted caloric intake induces a series of metabolic responses to nutrition deficiency, effective regulation of metabolic processes to adapt to this change could be another important mechanism underlying the effect of CR on longevity. One approach to interpreting CR in the regulation of metabolic pathways is through interventions to treat human obesity, which has become an important public health issue in recent years. Obesity is a common metabolic disorder characterized by excessively accumulated body fat and is closely related to a series of human diseases, including diabetes, hypertension, dyslipidemia, cardiovascular complications and even cancer, which are recognized causes of accelerated aging [64]. Therefore, obesity prevention could be a key underlying factor in the anti-aging effects of CR. Because of its substantial and promising effects in promoting weight loss, CR is widely used in clinical weight control intervention [65]. Current studies focusing on short-term CR interventions in obese human subjects revealed that hypocaloric diets cause DNA methylation changes in specific loci, such as *ATP10A*, *WT1 *and *TNF-α*, which could be used as early indicators of a response to the metabolic effects and as predictors of outcomes in weight-loss programs [66-68]. Although further CR studies have identified a pool of DNA methylation-controlled candidate genes that may be closely correlated with metabolic pathways, widespread methylation changes on numerous gene loci that facilitate CR in reprogramming the DNA methylation profile may also explain a powerful and universal effect of CR in influencing different aspects of human health. Thus, better understanding the functions of these DNA methylation-sensitive genes may contribute not only to optimizing personal weight-loss plans, but more important, to developing a novel application in slowing down of aging processes and the prevention of aging-related diseases.

Surprisingly few studies have investigated genome-wide alterations in DNA methylation profiles in CR-induced longevity using *in vivo *or *in vitro *models. Thus, the complete methylation-regulated pathways and target genes that may be responsible for CR-induced longevity remain unknown. Further investigations in this particular area show promising prospects in developing novel clinical preventative or therapeutic approaches to aging-related degenerative diseases.

### Effects of histone remodeling in control of aging during caloric restriction

**Figure 2:**
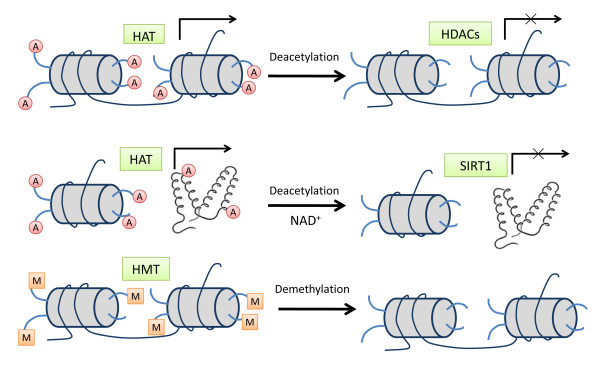
**Histone modification pathways**. Histone acetylation is mediated by HAT and deacetylation is catalyzed by the HDAC family. The upper row represents histone acetylation/deacetylation processes mediated by classic HDAC family members, including classes I, II and IV. Histone acetylation causes an open chromatin structure, leading to active transcription, whereas histone deacetylation is always associated with transcriptional repression. The middle row indicates a class III HDAC family member, SIRT1, which deacetylates both histone and protein substrates, resulting in gene silencing in most cases. The lower row represents histone methylation as another important histone modification. Histone methylation is mediated by HMT, and either gene activation or gene repression by histone methylation is dependent upon the particular lysine residue that is modified. HAT, histone acetyltransferase; HDAC, histone deacetylase; HMT, histone methyltransferase. SIRT1, Sirtuin (silent mating type information regulation 2 homolog) 1.

Histone modifications affect the basic structure of the chromatin unit, the nucleosome. The nucleosome consists of 146 bp of DNA wrapped around an octamer of histones (two copies of H2A, H2B, H3 and H4 monomers) [69]. In most cases, histone remodeling occurs at the N-terminal group of lysine (K) residues in histones by diverse modification patterns such as acetylation, methylation, ubiquitination and ADP ribosylation, among which histone acetylation or deacetylation changes are considered to be the most prevalent mechanisms of histone modifications [27]. Histone modifications are associated with both gene activation and gene repression. The combination of modifications within histone tails directly changes nucleosome configuration and results in the status of chromatin switching to either a compacted status (tight-close) or a relaxed status (loose-open) [70]. Therefore, histone modifications determine the level of openness of chromatin and thus the degree of gene activity within a certain DNA region. For example, a deacetylated histone lysine residue has the positive charge, which attracts the negatively charged DNA strand producing a compact chromatin state that is associated with transcriptional repression. By contrast, the modification of histone acetylation removes the positive charge and results in an open chromatin structure, which leads to active transcription (Figure 2).

#### Histone acetylation and deacetylation

Histone acetylation and deacetylation processes are catalyzed by specific enzymes called histone acetyltransferases (HATs) and HDACs, respectively [71,72] (Figure 2). At least four classes of the HDAC family have been identified: class I HDACs (HDAC1, HDAC2, HDAC3 and HDAC8) are most closely related to the yeast Rpd3 HDAC; class II HDACs (HDAC4, HDAC5, HDAC6, HDAC7, HDAC9 and HDAC10) share homology domains with the yeast enzyme Hda1; class III HDACs including Sirtuins 1, 2, 3, 4, 5, 6 and 7 are homologues of the yeast Sir2; and HDAC11 is the only member of class IV HDACs and closely related to the class I HDACs.

In addition to their deacetylation function, HDACs are believed to participate in the regulation of many cellular functions and gene expression through interactions with hundreds of different transcription factors [71,73]. It has also been reported that HDAC activity is increased during CR, suggesting that global deacetylation may be a protective mechanism against nutrition stress and may influence the aging processes [31].

We have found that altered binding enrichment of HDAC1, such as on the promoter regions of the *p16^INK4a ^*and human telomerase reverse transcriptase (*hTERT*) genes, the latter of which is a key determinant of telomerase activity closely associated with aging regulation, leads to beneficial expression changes of these two genes and contributes to longevity under CR conditions (Figure 1 and Table 2) [31,74,75]. Therefore, remarkable roles of the HDAC family in regulation of aging during CR highlight the potential application of related epigenetic drugs or clinical strategies in aging and aging-related diseases.

At this point, HDAC inhibitors have emerged as an exciting new class of potential anticancer agents despite little evidence pertaining to other aging-related diseases. HDAC inhibition causes acetylation of nuclear histones, leading to transcriptional activation of several key tumor-related genes, such as the cyclin-dependent kinase inhibitor *p21^WAF1/CIP1^*, *p53*, *GATA-1 *and *estrogen receptor-α*, which contribute to inhibiting cancer proliferation and inducing differentiation both *in vitro *and *in vivo *[76,77]. Several HDAC inhibitors with impressive antitumor activity and relatively low toxicity, such as depsipeptide, phenylbutyrate, valproic acid and suberoylanilide hydroxamic acid, are currently undergoing phases I and II clinical trials (Table 3) [78,79]. These structurally diverse molecules with properties of HDAC inhibition support a model in which HDACs are the critical cellular targets causing chromatin instability and tumorigenesis. Bioactive dietary ingredients, such as green tea polyphenols, broccoli sprouts and soybean genistein, that have natural HDAC inhibition properties are also considered as potential cancer chemoprevention compounds which are being studied in preclinical trials (Table 3) [62,63,80]. This may apply to aging-associated degenerative diseases that involve similar abnormalities such as tumorigenesis, and further studies are urgently needed in this area.

#### Sirtuin 1 and its substrates

Several HDAC families have been identified, including class III NAD^+^-dependent HDACs such as Sirtuin 1. Sirtuin 1 (SIRT1) in mammals, and its orthologs in other species (Sirtuin 2 in yeast), deserves special attention due to its fundamental impact on aging regulation and CR-related lifespan extension [32-36]. The unusual enzymatic activity of SIRT1, which largely depends on NAD/NADH ratio, a key indicator for oxygen consumption, respiratory chain and metabolic rate, suggests that this protein is tightly connected to the metabolic state of cells.

The promising effect of SIRT1 in mediating CR and lifespan extension is supported by a diverse range of animal models, human subjects and even *in vitro *CR cellular systems [31-33,35,36,81-85]. Activation of SIRT1 is frequently seen in different animal organs affected by CR, whereas inactivation of SIRT1 may lead to abolition of lifespan extension, suggesting a pivotal role of SIRT1 in lifespan regulation during CR. SIRT1 was initially discovered for its activation in response to CR and its role in the prolongation of lifespan in yeast [33]. This theory is solidified by findings in *Drosophila*, in which CR induces Sir2 activation and subsequent lifespan extension in wild-type *Drosophila *rather than in Sir2 mutants [33]. Further, either the Sir2 activator resveratrol or the overexpression of Sir2 leads to lifespan extension, and this longevity is not further induced by CR, suggesting that Sir2 is an important modulator in the regulation of aging processes.

In mammals, SIRT1-null mice do not survive longer, and most of them die during the postnatal period [86,87]. They exhibit growth retardation, multiple developmental defects and sterility, suggesting an important role of SIRT1 in early development. The role of endogenous SIRT1 in mammalian metabolic regulation has focused mainly on rodents in the context of fasting under the condition in which SIRT1 overexpression or its activity is upregulated [33-36]. Extensive studies have shown that CR induces SIRT1 expression in several tissues of mice or rats [33]. The potential mechanisms by which SIRT1 mediates CR-induced metabolic alterations and subsequent aging retardation primarily involve two aspects: first, SIRT1 activation increases stress resistance by negative regulation of proapoptotic factors such as p53 and Foxo (Table 2) [88-92]; second, SIRT1 causes a series of endocrine responses, including inhibition of adipogenesis and insulin secretion in pancreatic β cells by regulation of key metabolism-associated genes such as peroxisome proliferator-activated receptor γ coactivator 1α (*PGC-1α*) (Table 2) [93,94], which facilitates stress resistance and longevity (Figure 1).

In yeast, Sir2-mediated deacetylation of histones H3 and H4 and concomitant silencing of protein recruitment occurs specifically in heterochromatic regions located at extrachromosomal ribosomal DNA, telomeres and silenced mating-type loci, which benefit lifespan extension in yeast [33,57,83,95]. Human SIRT1 establishes and maintains chromatin silencing by preferential deacetylation at histone H4 lysine 16 (H4K16), but it also has been shown to deacetylate the loci of histone H3 lysine 9 (H3K9) *in vitro *[96] (Figure 2). Further, SIRT1 affects the levels of histone methylation by deacetylation of SUV39H1, a mammalian histone methyltransferase suppressor, leading to increased levels of the trimethylated H3K9 (H3K9Me3) modification (a chromatin repressor) [97,98].

Although classed as a HDAC, SIRT1 deacetylates a broad range of substrates, including many nonhistone substrates [33,83] (Table 2 and Figure 2). These potential substrates may include several key transcription factors and regulatory proteins that are involved in multiple pathways linked to physiological and metabolic processes that contribute to lifespan extension by CR (Table 2 and Figure 1). CR is known to exert its effect by inhibition of apoptosis, which is one of the most important regulatory mechanisms [14,25,26]. In this regard, p53 is notable due to its important role in the regulation of cell death and apoptosis. Downregulated p53 by SIRT1 deacetylation may affect lifespan by negatively regulating cellular apoptosis and replicative senescence processes [88-90]. Another important protein that influences apoptosis is Foxo. Foxo protein can be directly deacetylated by SIRT1 at lysine residues and its expression is reduced, thereby repressing Foxo-mediated apoptosis [91,92]. In addition, the DNA repair protein, Ku70, can become deacetylated by SIRT1, allowing it to inactivate the proapoptotic factor Bax, resulting in apoptosis inhibition [99,100].

SIRT1 can also regulate the expression of genes that are involved in metabolic pathways. PGC-1α represents the best example of these proteins in CR studies (Table 2). PGC-1α is a key regulator of gluconeogenesis and fatty acid oxidation [93,94]. It is activated by SIRT1-mediated deacetylation, which increases its ability to coactivate HNF4α, a transcription factor that promotes the expression of gluconeogenic genes and represses genes involved in glycolysis [34,83]. Therefore, SIRT1-induced changes in *PGC-1α * expression, and its downstream metabolic pathways provide a link between SIRT1 activation and the stimulation and response of metabolic systems under CR conditions.

Another key gene that can be epigenetically regulated by SIRT1 is *p16^INK4a^*, which is a cyclin-dependent kinase inhibitor linked to cellular senescence regulation [101] (Table 2). This gene was originally identified as an important tumor suppressor gene in that it negatively regulates the cell cycle and inhibits tumor growth [102,103]. Current studies show that *p16^INK4a ^*is significantly accumulated during the aging processes, indicating that *p16^INK4a ^*can serve as a robust aging biomarker [104,105]. Our recent studies using human cells show that CR-activated SIRT1 can directly bind to the *p16^INK4a ^*promoter and decrease its expression through a deacetylation effect, which contributes to delaying the aging process and to lifespan extension [84]. Therefore, SIRT1, acting as a nutrition sensor, decodes the nutrition flux to ensure homeostasis or even a beneficial state such as increased longevity by reorganizing the global chromatin structure and dynamically epigenetically regulating specific genes that may involve apoptosis regulation, metabolic control and cellular senescence. Besides its pronounced roles in regulating epigenetic processes, SIRT1 has been well demonstrated to regulate genes and interact with signaling other than epigenetic control during CR, suggesting that SIRT1 may play an important role in multiaspect cross-talk between epigenetic and genetic pathways.

#### Histone methylation

Besides histone acetylation, histone methylation is another important histone modification that regulates gene expression [72] (Figure 2). In contrast to histone acetylation, which is always associated with open chromatin status and subsequent gene activation, differentially methylated forms of histones show unique association patterns with specific proteins that recognize these markers and thus lead to gene silencing or activating effects. Lysine residues on histones can be mono-, di- or trimethylated, and either activation or repression is dependent upon the particular lysine residue that is modified [106,107]. Our current studies have shown that histone methylation modifications such as di- or trimethylated histone H3 at lysine residue 3 or 4 can also regulate expression changes of key aging-related genes, including *p16^INK4a ^*and *hTERT*, thereby contributing to CR-induced lifespan extension of human cells (Figure 1 and Table 2) [31,84]. In other studies, researchers have reported that *p16^INK4a ^*expression can be regulated by H3K27 trimethylation, which serves as a recruitment signal for BMI1-containing polycomb-repressive complexes such as PRC1 during cellular senescence [108-110]. Therefore, the status of specific histone methylation can also serve as a transcription modulator by interacting with different transcription factors and regulate aging processes under CR conditions.

### Potential epigenetic treatments for aging-related diseases

The promising impact of the chromatin regulators on aging interference provides an excellent opportunity to prevent for human aging-related diseases by applying potential epigenetic drugs. An example of this is resveratrol, a natural compound found in grapes and red wine which has been demonstrated to extend lifespan in *Saccharomyces cerevisiae*, *Caenorhabditis elegans *and *Drosophila *through remodeling chromatin structure via mediation of SIRT1 activity [111-113]. It has been reported that resveratrol can activate SIRT1 mechanisms and mimic SIRT1-induced CR cascades, leading to increased longevity [114]. In addition to its effect on longevity, this compound is known to positively influence metabolism and reduce fat and glucose levels, resulting in increasing glucose tolerance and activation of several signaling pathways that are relevant to antistress, antioxidation and increased mitochondrial biogenesis [115,116]. These effects were illustrated by a current finding showing that resveratrol opposes the effects of a high-fat diet in mice [117]. Due to the toxicity of the high-fat diet, control animals in this study had early mortality, whereas resveratrol improved the health and survival rate of these mice, suggesting the important role of resveratrol in the aging process. Clinically, a total of 31 human studies involving resveratrol have been reported in the US national database http://clinicaltrials.gov/. These studies aimed at investigating the potential role of resveratrol in diabetes, obesity, Alzheimer's disease and cancer (Table 3). These studies have revealed promising and universal effects of resveratrol by favorably altering cell proliferation, increasing cellular detoxification, protecting DNA damage, modulating metabolic processes and inhibiting tumorigenesis, which significantly improve human health and lead to increased human lifespan [118,119].

Epigenetic therapy has shown powerful clinical potential in delaying aging and preventing aging-related diseases, especially cancer. As we have discussed previously, DNMT inhibitors, inlcuding azacitidine and decitabine, as well as HDAC inhibitors, such as depsipeptide, phenylbutyrate, valproic acid and suberoylanilide hydroxamic acid, have been widely used for cancer treatment in both experimental studies and clinical trials (Table 3). Studies have also indicated that resveratrol is a potent cancer chemopreventative agent. These findings are extremely encouraging, and future studies focusing on development of novel epigenetic drugs are urgently needed to develop effective clinical strategies to treat human aging-related diseases [120].

### "Epigenetic diets" that mimic the effects of caloric restriction on lifespan

The significant epigenetic impact of CR on delaying aging and preventing aging-related diseases has motivated efforts to identify natural or synthetic compounds that mimic the effects of CR. A broad range of diets have been identified that mediate epigenetic processes, the so called "epigenetic diets," providing potential to reduce aging-associated disease incidence and possibly extending the quality and length of the human lifespan by simple consumption of such diets or extracted bioactive dietary compounds [121] (Table 3). As described previously, resveratrol represents an excellent example of an "epigenetic diet" and acts as a SIRT1 mimic that leads to increased longevity *in vivo *and *in vitro *[111-119]. Other important epigenetic diets have recently been identified, such as green tea, broccoli sprouts and soybeans, and the bioactive compounds extracted from these diets have received extensive attention due to their profound effects on cancer prevention by altering the aberrant epigenetic profile in cancer cells [62,63,80,122-125]. In particular, long-term consumption of these epigenetic diets is highly associated with a low incidence of various aging-related degenerative diseases such as cancer and cardiovascular disease, suggesting that these bioactive diets may affect aging processes by altering chromatin profiles that also occur in CR [2]. For instance, global gene expression profiling can be used to identify useful compounds correlated with biological age. Dhahbi *et al. *[126] developed gene expression profiling methods to discover potential pharmaceuticals capable of mimicking the effects of CR, which may open a new avenue in the discovery of promising candidates that mimic CR and delay aging.

## Conclusions

Epigenetically mediated changes in gene expression have become a major molecular mechanism linking CR with its potential for improving cell function and health throughout the life course, leading to delaying the aging processes and extending longevity. Understanding the epigenetic mechanisms that influence the nature of aging by CR might lead to discoveries of new clinical strategies for controlling longevity in humans. As discussed in this review, two primary epigenetic codes, DNA methylation and histone modification, play important roles in regulating chromatin structure and expression of key genes to elicit the global response to CR (Figure 1). The readily reversible feature of epigenetic alterations provides great potential for the use of specific interventions aimed at reversing epigenetic changes during aging, which may have a significant impact on delaying aging and preventing human aging-associated diseases. Although our knowledge of the role of epigenetic mechanisms in CR and its related health impact is relatively limited at present, further studies will likely provide more precise interpretation of this complicated interaction, thereby facilitating the discovery of novel approaches linking dietary or pharmaceutical interventions to human longevity. We have learned of the profound effects of SIRT1 and its mimics, such as resveratrol, in influencing aging processes, and this exciting example implies that the key to improving the quality of human life, especially for senior citizens, is in the not too distant future.

## Abbreviations

bp: base pair; CR: caloric restriction; DNMT: DNA methyltransferase; HDAC: histone deacetylase; HAT: histone acetyltransferase; *hTERT*: human telomerase reverse transcriptase.

## Competing interests

The authors declare that they have no competing interests.

## Authors' contributions

YL wrote the first draft of the manuscript. All other authors contributed to the development of the manuscript. All authors read and approved the final manuscript.

## Pre-publication history

The pre-publication history for this paper can be accessed here:

http://www.biomedcentral.com/1741-7015/9/98/prepub
